# Analysis of Three-Way Game of Straw Return System under the Green Transformation of Agriculture

**DOI:** 10.3390/ijerph20054520

**Published:** 2023-03-03

**Authors:** Linling Geng, Li Zhou, Yifeng Zhang

**Affiliations:** School of Economics and Management, Nanjing Forestry University, Nanjing 210037, China

**Keywords:** straw returning, multiple subjects, synergy, tripartite game

## Abstract

Open burning of straw is the most significant problem of environmental pollution in rural areas. Returning straw to the fields is beneficial to rural environmental management and rural development. Comprehensive utilization of straw in the field not only reduces environmental pollution, but also benefits food production and farmers’ income. Because planting farmers, enterprises, and local governments have different interests, it is difficult for the straw return system to operate soundly. In this study, a three-party evolutionary game model of farmers, enterprises, and local governments was constructed to analyze the evolutionary stability of the strategic choices of the three subjects, explore the influence of each element on the strategic choices of the three parties, and use Matlab2022b simulation to further analyze the dynamic evolution of the game behavior of the system subjects under the given benefits and the given subjects, respectively. The study findings showed that the higher the preferences given by the local government, the higher the probability of farmers and enterprises participating in the straw return system. Only with the participation of local governments can the straw return system be operated robustly. Our study findings also revealed that the interests of farmers must be fully protected in order to mobilize the main body and stimulate market dynamics. The overall findings of this study provide useful insights for promoting government agencies to govern the local environment, increase local revenues, and build integrated waste utilization systems.

## 1. Introduction

Arable land is a basic resource for maintaining sustainable and high-quality food production, but, at present, China’s arable land is concentrated with problems such as acidification of arable land in the south, salinization of arable land in the north, and degradation of black land in the northeast [1]. As a biological resource rich in organic matter, crop straw can be scientifically and effectively returned to the field to improve soil structure, increase soil organic matter, and promote the development of crop roots [2]. The open burning of large amounts of crop straw produces harmful gases that pollute the environment. In response to the goal of achieving carbon peaking by 2030 and carbon neutrality by 2060, as well as protecting the safety of arable land, the central government has actively introduced several policies to return straw to the fields. The effectiveness of the policy implementation needs to be further improved, with the following three main issues. For one thing, local governments are supposed to take responsibility for local environmental management, but they are often more willing to invest in short-term projects that bring local economic growth due to the pressure of economic performance assessment in the short term [3]. The supervision and incentives for farmers to return their straw to the fields need to be borne by local coffers, and relying on the government alone for continued blood transfusions is not a long-term solution [4]. Second, farmers are both the main body of agricultural production and environmental protection, and they should be fully motivated. In areas with tight rice and wheat stubble, the sowing and harvesting sessions operate on a tight schedule, making it difficult for farmers to take time out of the small amount of leisure they have for straw collection [5]. Straw burning behaviors have a clear externality effect, and there is free-riding by farmers, which both lead to weak motivation [6]. Third, straw fueling, basing, and raw materialization as new technology requires companies to invest in more research and development costs and advanced production equipment [7]. Straw collection and storage are dominated by enterprises, greatly increasing transport and storage costs. Therefore, we must give full play to market dynamics, cultivate and grow straw utilization market players, and explore the establishment of a scalable and sustainable industrial development model and efficient straw utilization system [8].

Specifically, on the government side, the local government is both the implementer of the straw return policy and a rational person seeking to maximize its economic interests, and the behavior of the local government is a key factor in the effective implementation of the straw return policy [9]. Local governments compete not only for economic and tax revenues but also for political promotion, and one official’s improved chances of promotion mean another’s reduced chances of promotion, creating a zero-sum game. The government faces tax budget constraints and difficulties in reconciling cross-regional ecological cooperation [10]. In the context of underdeveloped rural markets, rules for the functioning of acquaintance society are more prevalent [11]. The intervention of village governments is an exogenous force, difficult to embed in the village society of acquaintances, and interventions far from the masses are often ineffective and costly [12]. We need to make good use of the familiar attributes of village cadres and village grassroots organizations in rural society, and use their appeal to guide farmers to participate in straw return [13]. On the farmer side, straw disposal is essentially an economic act, and cost–benefit is a key factor in determining farmers’ adoption of straw return [14]. Scientific straw return can fix organic carbon in the soil, improve soil structure, increase soil fertility, and replace the input of chemical fertilizer elements [15]. However, straw is large and numerous and relies on mechanical operations, reducing labor costs and increasing machinery costs [16]. Other things being equal, with rising fertilizer and labor factor prices and increased machinery ownership, farmers are more willing to participate in straw return [17]. The initial benefits of returning straw to the field are not obvious and affect the willingness of farmers to adopt it. Problems such as unsupported and immature agro-technology require the government to establish a reasonable compensation mechanism [18]. Compensation is based on the subject of farmers, accounting for compensation standards based on the function of crop carbon sinks, and creating a variety of compensation standards and forms [19,20,21]. On the enterprise side, the production activities of straw recycling and processing enterprises rely on a better business environment driven by national policies [22]. However, the straw return policy supports more subsidies in the straw return process, and the cost of the straw leaving the field, collection, and transport is still borne by enterprises. We can clarify the names of specific products such as agricultural waste resourceization that are given tax incentives to greatly motivate enterprises and stimulate market dynamics [23]. Most scholars have measured the performance of agricultural waste recycling, and the results all show a low level, mainly due to the inefficient scale of factor inputs [24,25].

Straw return not only has ecological benefits but also economic benefits, but its externality makes the private benefit cost of straw return not equal to the social benefit cost, making it difficult to achieve Pareto optimality, so game theory needs to be used to analyze the strategic choices between interest players. The central government acts to protect the ecological environment and maintain sustainable development, standing at the starting point of maximizing the interests of society as a whole, while local governments seek to maximize their performance promotion and farmers seek to maximize their interests, with different interests and different behavioral choices [26].

Several scholars have constructed game models of various actors in the rural environment and analyzed their equilibrium strategies. From the perspective of game research, Liu et al. and Xu et al. [27,28] focused on the game between the central government and local governments as well as the game between farmers and local governments. They concluded that the supervision of the central government is in direct proportion to the probability of local government violations. The ecological protection behavior of farmers is affected by the probability of violation by local governments. Hao et al. [29] focused on the game between ecological suppliers and ecological consumers. They concluded that no matter whether the ecological supplier chooses to protect or not, the ecological consumer chooses not to compensate. Only when the government increases the punishment can the balance be reached. From the perspective of tripartite game research, Asian et al. [30] focused on the tripartite game between the industrial management department, the technology promotion department, and the farmers and adopted the DEA-HR model to draw the conclusion that the collaborative innovation ability of the participants in the matrix recycling of agricultural waste is in a “coordinated and generally effective” state. There are differences in interest demands and information asymmetry among participants, which restrict the development of matrix industries. Zhang et al. [31] focused on the game between the government, farmers, and contractors, and they concluded that the government plays a guiding role in the behavior strategies of farmers and contractors, that farmers’ transfer of farmland is a prerequisite for the system to become stable, and that farming cost is also an important factor affecting the three parties to reach a stable equilibrium point. From the perspective of the inter-regional game, Liu et al. [32] constructed the game between the eastern and central and the western regions, and they found that the regional unbalanced development strategy led to the unfair allocation of farmland resources nonagricultural indicators, and that the eastern region took the lead in implementing farmland nonagricultural transformation, which caused pressure and difficulties for farmland protection in the central and western regions. Table 1 organizes these views.

In summary, there is still room for research on rural environmental governance. First, scholars have focused more on the broad areas of arable land, watersheds, and waste, ignoring the uniqueness of straw returning to the land. Second, the existing literature has fully analyzed the game relationships between governments, between governments and farmers, between enterprises and farmers, and between regions, focusing on the guiding role of governments and neglecting the role of market regulation. Finally, the parties to the game often calculate costs taking into account only short-term and long-term material costs, and do not include the shadow wages of farmers as a cost. We give full play to the role of market mechanisms in waste management, encourage farmers to market their waste, and promote the marketization of waste collection, storage and transportation, organic fertilizer, and other related industries. The government supports the construction of various waste processing and distribution centers, as well as the improvement of storage and logistics facilities.

To this end, the following attempts will be made in this paper. First, rural waste management is refined to the micro perspective of straw return to the fields. This provides a more specific analysis of the evolutionary game process of all stakeholders in the implementation of straw return. We use feedback dynamic complexity analysis theory to carry out theoretical innovation research on rural straw utilization, and then feed back the innovative theory to guide the construction of a comprehensive straw utilization system, repeatedly, to achieve independent innovation in management theory. Second, we will land the main players of the straw return game on enterprises, farmers, and the government, build a system guided by the government, regulated by the market, and actively participated in by farmers, and analyze the stability and equilibrium of the straw comprehensive use system. Third, we take into account the farmer’s shadow wage when the farmer is sowing and planting. Because straw collection and leaving the field take up the farmer’s leisure, we calculate the farmer’s shadow wage, i.e., leisure in this case, as part of the farmer’s costs.

The remainder of the paper is organized as follows: Part Ⅱ is the hypotheses and model, which constructs a three-way game model between firms, farmers, and the government. Part Ⅲ presents the results of the model derivation. This section presents the inferences and mathematical proofs based on the model. Part Ⅳ is the simulation analysis. This section analyzes the dynamic evolution of each player with specific examples. The fifth part is the conclusion.

## 2. Hypotheses and Model

### 2.1. Hypotheses

We propose the following hypotheses to construct a game model to analyze the stability of the three-way strategy and equilibrium point between firms, farmers, and the government, as well as the influential relationships. The meaning of the specific parameters is shown in Table 2.

**Hypothesis 1.**  *Straw comprehensive utilization enterprises are system participant 1, farmers are system participant 2, and local governments are system participant 3. All three parties are economically rational and seek to maximize their interests, and their strategic choices gradually converge to equilibrium over time.*

**Hypothesis** **2.**  *Strategic space for integrated culm utilization companies*$\alpha =\left(\alpha_{1},\alpha_{2}\right)$*= (valorizing, no valorizing), the probability of choosing production is x, the probability of not producing is 1 − x,*x∈[0,1]*; strategic space for growing farmers*$\beta =\left(\beta_{1},\beta_{2}\right)$*= (valorizing, no participation), the probability that a farmer chooses to participate is y, and the probability that he does not participate is 1 − y,*y∈[0,1]*; strategic space for local government*$\gamma =\left(\gamma_{1},\gamma_{2}\right)=\left(subsidized,\;no\;subsidized\right)$*, the probability that the government will choose to subsidize z, the probability of subsidy is 1 − z,*z∈[0,1].

**Hypothesis** **3.**  *The annual revenue of the enterprise from normal production and operation is*$\;R_{p}$*, the cost of straw processing is*$C_{1}$*, and the cost of no straw processing is*$C_{2}$*. If companies reprocess straw, they need to increase their research and development costs and have equipment sufficient for production as well as storage facilities, whereas if they produce the original goods, they simply produce them under the original production conditions; therefore,*$C_{1}>C_{2}$*. Companies choosing to produce straw conversions can receive tax incentives or subsidies from the government*$D_{x}$*; however, if companies choose not to produce, resulting in untreated straw, environmental pollution can harm the company and entail potential ecological costs*$\;B_{x}$*,*$C_{1}-C_{2}>B_{x}$.

**Hypothesis** **4.**  *The normal annual return to the grower from the operation of the farming activity is*$\;V_{1}$*. If farmers are involved in returning straw to the field, subsidized by the government*$D_{y}$*, revenue from sales to manufacturing companies*$V_{2}$*; however, straw collection from the field costs farmers’ leisure E. If farmers do not participate in straw return, they do not receive government subsidies or income from selling to businesses, and there are potential ecological costs to the environment caused by the land environment*$\;B_{y}$.

**Hypothesis** **5.** Annual performance gains for local governments are A, if the local government provides financial subsidies for straw return, then the farmers’ subsidies are $D_{y}$*, preferential production practices for businesses* $D_{x}$*, if the local government does not provide subsidies, the enthusiasm of the main body to participate in environmental governance is not high. The potential ecological cost of this part is* $B_{z}$*. The ecological costs borne by local governments are greater than the concessions or subsidies given to enterprises and farmers, i.e.,*$\;B_{z}>D_{x}$*,* $B_{z}>D_{y}$.

### 2.2. Model

Based on the above hypotheses, a pure strategy game matrix is constructed for firms, growers, and local governments, as shown in Table 3.

## 3. Results

### 3.1. The Strategic Stability of the Company

The expected returns and average expected returns for firms choosing production and nonproduction strategies are specified as follows. $E_{x1}$ is the expected income of company participation, $E_{x2}$ is the expected income that the company does not participate in, $\;\bar{E_{x}}$ is the average expected income.

(1) $$E_{x1}=yz\left(R_{p}-C_{1}+D_{x}\right)+y\left(1-z\right)\left(R_{p}-C_{1}\right)+\left(1-y\right)z\left(R_{p}-C_{1}+D_{x}\right)+\left(1-y\right)\left(1-z\right)\left(R_{p}-C_{1}\right)$$

(2) $$E_{x2}=yz\left(R_{p}-C_{2}-B_{x}\right)+y\left(1-z\right)\left(R_{p}-C_{2}-B_{x}\right)+\left(1-y\right)z\left(R_{p}-C_{2}-B_{x}\right)+\left(1-y\right)\left(1-z\right)\left(R_{p}-C_{2}-B_{x}\right)$$

(3) $$\bar{E_{x}}=xE_{x1}+\left(1-x\right)\;E_{x2}$$

The dynamic replication equation for the proportion *x* that the firm chooses to produce and the first-order derivative are obtained as

(4) $$F\left(x\right)=\frac{dx}{dt}=x\left(E_{x1}-\bar{E_{x}}\right)=x\left(x-1\right)\left(C_{1}-zD_{x}-C_{2}-B_{x}\right)$$

(5) $$\frac{d\left(F\left(x\right)\right)}{dx}=\left(2x-1\right)\left(C_{1}-zD_{x}-C_{2}-B_{x}\right)$$

(6) $$\mathrm{Let}\;G\left(z\right)=C_{1}-zD_{x}-C_{2}-B_{x}$$

**Figure 1:**
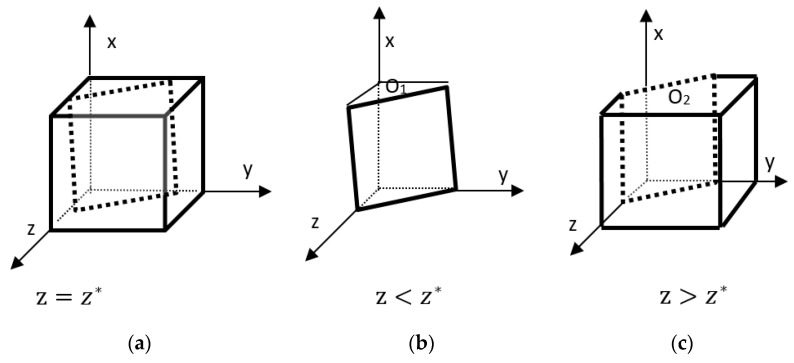
Phase diagram of the evolution of corporate strategy. (**a**) Phase diagram when z = z *; (**b**) Phase diagram when *z* < z *; (**c**) Phase diagram when *z* > z *.

According to the stability theorem for differential equations, the probability that a firm chooses to produce in a steady state must satisfy: F(x)=0 and $\frac{d\left(F\left(x\right)\right)}{dx}<0$. Due to $\frac{d\left(G\left(z\right)\right)}{dz}=-D_{x}$, of which $D_{x}>0$; therefore, G(z) is a monotonically decreasing function. $z=z^{*}=\frac{C_{1}-C_{2}-B_{x}}{D_{x}}$, G(z)=0, $\frac{d\left(F\left(x\right)\right)}{dx}\equiv 0$; this is unable to determine the steady state. $\;z>z^{*}$, G(z)<0, $\frac{d\left(F\left(x\right)\right)}{dx}{|}_{x=0}>0$; $z<z^{*}$, G(z)>0, $\frac{d\left(F\left(x\right)\right)}{dx}{|}_{x=0}<0$. $\;z>z^{*},\;\;G\left(z\right)<0$, $\frac{d\left(F\left(x\right)\right)}{dx}{|}_{x=1}<0$. $\;z<z^{*},\;\;G\left(z\right)>0$, $\frac{d\left(F\left(x\right)\right)}{dx}{|}_{x=1}>0$. Therefore, $z<z^{*}$, x=0 is the firm’s evolutionary stability strategy. $\;z>z^{*}$, x=1 is the firm’s evolutionarily stable strategy. The evolutionary phase diagram of the specific firm’s strategy is shown in Figure 1 below.

As shown in Figure 1, the volume for which the firm has a stable nonproduction probability of $O_{1}$ is $V_{O_{1}}$, and the volume for which the stable production probability of $O_{2}$ is $V_{O_{2}}$ is calculated as follows.

(7) $$V_{O_{1}}=\int_{0}^{1}\int_{0}^{C_{1}-C_{2}-B_{x}}\frac{C_{1}-C_{2}-B_{x}}{D_{x}}dxdy=\frac{{\left(C_{1}-C_{2}-B_{x}\right)}^{2}}{D_{x}}$$

(8) $$V_{O_{2}}=1-V_{O_{1}}=1-\frac{{\left(C_{1}-C_{2}-B_{x}\right)}^{2}}{D_{x}}$$

**Corollary 1.**  The probability that a firm will produce is positively correlated with government preferences and ecological costs. The more preferential subsidies given by the government and the greater the ecological costs borne, the greater the probability that the firm will produce. The probability of producing is negatively related to the difference between the cost of producing and not producing, meaning that the more a company spends on producing straw-processed goods, the less likely it is to choose to produce.

**Proof 1.**  $\frac{\partial V_{O_{2}}}{\partial D_{x}}=\frac{{\left(C_{1}-C_{2}-B_{x}\right)}^{2}}{D_{x}{}^{2}}>0$; $C_{1}-C_{2}-B_{x}>0$ as mentioned in the previous assumption 3, $\frac{\partial V_{O_{2}}}{\partial B_{x}}=\frac{2(C_{1}-C_{2}-B_{x})}{D_{x}}>0$; $\frac{\partial V_{O_{2}}}{\partial (C_{1}-C_{2})}=-\frac{\left(C_{1}-C_{2}-B_{x}\right)}{D_{x}}<0$. In summary, an increase in $D_{x}$, an increase in $B_{x}$, and a decrease in $C_{1}-C_{2}$ all increase the probability of a firm’s stable production of straw-processed commodities. □

Corollary 1 suggests that local governments can provide policy incentives or subsidies as appropriate [33]. To reduce R&D costs, the government needs to establish an information sharing platform. To reduce the cost of transportation and development, the government should improve transport and storage infrastructure. The government should increase awareness of environmental protection and deepen the understanding of the damage caused by environmental pollution to enterprises, and should guide them to actively engage in the processing of straw in production [34].

### 3.2. The Strategic Stability of Planting Farmers

The expected returns and the average expected returns for growers participating and not participating in straw returns are expressed specifically as follows. $E_{y1}$ is the expected income of farmers’ participation, $E_{y2}$ is the expected income that farmers do not participate in, $\bar{E_{y}}$ is the average expected income.

(9) $$E_{y1}=xz\left(V_{1}+V_{2}+D_{y}-E\right)+x\left(1-z\right)\left(V_{1}+V_{2}-E\right)+\left(1-x\right)z\left(V_{1}+D_{y}-E\right)+\left(1-x\right)\left(1-z\right)\left(V_{1}-E\right)$$

(10) $$E_{y2}=xz\left(V_{1}+E-B_{y}\right)+x\left(1-z\right)\left(V_{1}+E-B_{y}\right)+\left(1-x\right)z\left(V_{1}+E-B_{y}\right)+\left(1-x\right)\left(1-z\right)\left(V_{1}+E-B_{y}\right)$$

(11) $$\bar{E_{y}}=yE_{y1}+\left(1-y\right)\;E_{y2}$$

The dynamic replication equation for the proportion y involved in the grower’s strategic choice, and the first-order derivative, is

(12) $$F\left(y\right)=\frac{dy}{dt}=y\left(E_{y1}-\bar{E_{y}}\right)=y\left(y-1\right)\left(2E-B_{y}-xV_{2}-zD_{y}\right)$$

(13) $$\frac{d\left(F\left(y\right)\right)}{dy}=\left(2y-1\right)\left(2E-B_{y}-xV_{2}-zD_{y}\right)$$

(14) $$\mathrm{Let}H\left(x,z\right)=2E-B_{y}-xV_{2}-zD_{y}$$

According to the stability theorem of the differential equation, the probability that the grower chooses to participate in a steady state must satisfy:  F(y)=0 and $\frac{d\left(F\left(y\right)\right)}{dy}<0$. $\frac{\partial H\left(x,z\right)}{\partial x}=-V_{2}<0$; therefore, *H(x,z)* is a monotonically decreasing function concerning *x*.$\;x=x^{*}=\frac{2E-B_{y}-zD_{y}}{V_{2}}$, H(x,z)=0, $\frac{d\left(F\left(y\right)\right)}{dy}\equiv 0$. This does not determine the steady state. $x>x^{*}$, H(x,z)<0, $\frac{d\left(F\left(y\right)\right)}{dy}{|}_{y=0}>0$. $x<x^{*}$, H(x,z)>0, $\frac{d\left(F\left(y\right)\right)}{dy}{|}_{y=0}<0$. $x>x^{*}$, H(x,z)<0, $\frac{d\left(F\left(y\right)\right)}{dy}{|}_{y=1}<0$. $\;x<x^{*}$, H(x,z)>0, $\frac{d\left(F\left(x\right)\right)}{dx}{|}_{y=1}>0$. Therefore, $x<x^{*}$, y=0 is the evolutionary stabilization strategy for the selected participating cropping farmers. $\;x>x^{*}$, y=1 is the evolutionarily stable strategy for selecting the participating planting farmers. The evolutionary phase diagram of the specific planting farmer’s strategy is as follows.

**Figure 2:**
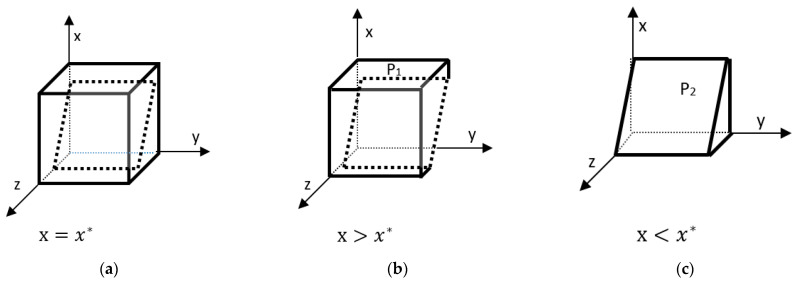
Phase diagram of the evolution of the grower’s strategy. (**a**) Phase diagram when *x* = x *; (**b**) Phase diagram when *x* < x *; (**c**) Phase diagram when *x* > x *.

As can be seen in Figure 2, the probability that a grower chooses to participate in straw return is $P_{1}$ and the volume is $V_{P_{1}}$, and the probability that a grower chooses not to participate in straw return is $P_{2}$ and the volume is $V_{P_{2}}$, with the following expressions.

(15) $$V_{P_{2}}=\int_{0}^{1}\int_{0}^{\frac{2E-B_{y}}{D_{y}}}\frac{2E-B_{y}-zD_{y}}{V_{2}}dydz=\frac{{\left(2E-B_{y}\right)}^{2}}{V_{2}D_{y}}-\frac{{\left(2E-B_{y}\right)}^{2}}{2V_{2}D_{y}}=\frac{{\left(2E-B_{y}\right)}^{2}}{2V_{2}D_{y}}$$

(16) $$V_{P_{1}}=1-V_{P_{2}}=1-\frac{{\left(2E-B_{y}\right)}^{2}}{2V_{2}D_{y}}$$

**Corollary 2.**  The probability of planting farmers’ participation is positively correlated with government subsidies and straw income. When the ecological cost of not participating in straw return is equal to the price of farmers’ leisure, then the probability of participating is negatively correlated with leisure and positively correlated with ecological cost. The more leisure a farmer has, the cheaper the price of leisure, and the higher the probability of participation in straw return. The higher the ecological cost, the higher the probability of participation.

**Proof 2.**  The partial derivatives of $D_{y}$, $V_{2}$, E, $B_{y}$ were obtained based on the expression of the probability of participation of the planting farmer, $V_{P_{1}}$. $\;\frac{\partial V_{P_{1}}}{\partial D_{y}}=\frac{{\left(2E-B_{y}\right)}^{2}}{2V_{2}}*\frac{1}{D_{y}{}^{2}}>0$, $V_{P_{1}}$ is positively correlated with $D_{y}$. $\frac{\partial V_{P_{1}}}{\partial V_{2}}=\frac{{\left(2E-B_{y}\right)}^{2}}{2D_{y}}*\frac{1}{V_{2}{}^{2}}>0$, $V_{P_{1}}$ is positively correlated with $V_{2}$. $\;B_{y}<2E$, $\frac{\partial V_{P_{1}}}{\partial E}=-\frac{1}{2V_{2}D_{y}}*4\left(2E-B_{y}\right)=\frac{2}{V_{2}D_{y}}*\left(B_{y}-2E\right)<0$, $V_{P_{1}}$ is negatively correlated with *E*. $\;\frac{\partial V_{P_{1}}}{\partial B_{y}}=\frac{1}{2V_{2}D_{y}}*2\left(2E-B_{y}\right)=\frac{1}{V_{2}D_{y}}*\left(2E-B_{y}\right)>0$, $V_{P_{1}}$ is positively correlated with $B_{y}$. Therefore, increasing $D_{y}$, $V_{2}$, $B_{y}$ and decreasing *E* all increase the probability of planting farmers’ participation in straw return. □

Corollary 2 suggests that local governments can increase subsidies for farmer participation behavior to ensure that farmers benefit from it. During the busy farming season, straw return to the fields is outsourced to socialized service organizations to reduce the use of expensive leisure costs [35]. We need to increase environmental awareness so that farmers realize that straw return can enhance land fertility, increase agricultural yields, and reduce the amount of fertilizer inputs to the land [36].

### 3.3. Strategic Stability Analysis of Local Governments

Expected returns with and without local government subsidies and the average expected return are expressed as follows. $E_{z1}\;$is the expected income of government participation, $E_{z2}$ is the expected income that the government does not participate in, and $\bar{E_{z}}$ is the average expected income.

(17) $$E_{z1}=xy\left(A-D_{x}-D_{y}\right)+x\left(1-y\right)\left(A-D_{x}-B_{y}\right)+\left(1-x\right)y\left(A-D_{y}-B_{x}\right)+\left(1-x\right)\left(1-y\right)\left(A-B_{x}-B_{y}\right)$$

(18) $$E_{z2}=xy\left(A-B_{z}\right)+x\left(1-y\right)\left(A-B_{y}-B_{z}\right)+\left(1-x\right)y\left(A-B_{x}-B_{z}\right)+\left(1-x\right)\left(1-y\right)\left(A-B_{x}-B_{y}-B_{z}\right)$$

(19) $$\bar{E_{z}}=zE_{z1}+\left(1-z\right)\;E_{z2}$$

The replicated dynamic equation for the proportion z of the government-selected strategy selection subsidy and its first-order derivative is as follows.

(20) $$F\left(z\right)=\frac{dz}{dt}=z\left(E_{z1}-\bar{E_{z}}\right)=z\left(z-1\right)\left(xD_{x}+yD_{y}-B_{z}\right)$$

(21) $$\frac{d\left(F\left(z\right)\right)}{dz}=\left(2z-1\right)\left(xD_{x}+yD_{y}-B_{z}\right)$$

(22) $$\mathrm{Let}\;I\left(x,y\right)=xD_{x}+yD_{y}-B_{z}$$

According to the stability theorem of the differential equation, the probability of the government choosing the subsidy to be in a steady state must satisfy: F(z)=0 and $\frac{d\left(F\left(z\right)\right)}{dz}<0$. $\;\frac{\partial I\left(x,y\right)}{\partial y}=D_{y}>0$; therefore, I(x,y) is a monotonically increasing function about *y*. $\;y=y^{*}=\frac{B_{z}-xD_{x}}{D_{y}}$, I(x,y)=0, $\frac{d\left(F\left(z\right)\right)}{dz}\equiv 0$. This does not determine the steady state. $y>y^{*},\;I\left(x,y\right)>0$, $\frac{d\left(F\left(z\right)\right)}{dz}{|}_{z=0}<0$. $y<y^{*}$, I(x,y)<0, $\frac{d\left(F\left(z\right)\right)}{dz}{|}_{z=0}>0$. $y>y^{*}$, I(x,y)>0, $\frac{d\left(F\left(z\right)\right)}{dz}{|}_{z=1}>0$. $y<y^{*}$, I(x,y)<0, $\frac{d\left(F\left(x\right)\right)}{dx}{|}_{z=1}<0$. Therefore, $y<y^{*}$, *z* = 1 is the evolutionary stabilization strategy for fertilizer producers. $y>y^{*}$, *z* = 0 is the evolutionary stabilization strategy for fertilizer producers. The evolutionary phase diagram of the local government selection strategy is shown below.

**Figure 3:**
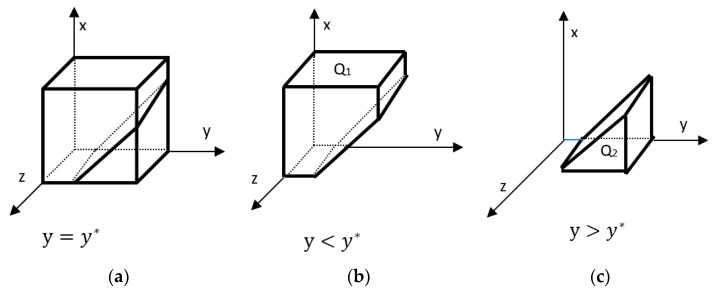
Phase diagram of the evolution of local government strategies. (**a**) Phase diagram when *y* = y *; (**b**) Phase diagram when *y* < *y* *; (**c**) Phase diagram when *y* > *y* *.

As shown in Figure 3 the probability that the local government chooses to subsidize and preferentially return straw to the field is $Q_{2}$ and the volume is $V_{Q_{2}}$, and the probability that it chooses not to subsidize is $Q_{1}$ and the volume is $V_{Q_{1}}$, as shown in the following expressions.

(23) $$V_{Q_{2}}=\int_{0}^{1}\int_{0}^{\frac{B_{z}-D_{y}}{D_{x}}}\frac{B_{z}-xD_{x}}{D_{y}}dzdx=\frac{B_{z}\left(B_{z}-D_{y}\right)}{D_{y}D_{x}}-\frac{{\left(B_{z}-D_{y}\right)}^{2}}{2D_{y}D_{x}}=\frac{B_{z}{}^{2}-D_{y}{}^{2}}{2D_{y}D_{x}}$$

(24) $$V_{Q_{1}}=1-V_{Q_{2}}=1-\frac{B_{z}{}^{2}-D_{y}{}^{2}}{2D_{y}D_{x}}$$

**Corollary 3.**  The probability that local governments choose to subsidize farmers is negatively correlated with fiscal expenditures and positively correlated with ecological costs.

**Proof 3.**  According to the expression of the local government subsidy probability $V_{Q_{2}}$, the partial derivatives of $D_{x}$*,* $D_{y}$*,* $B_{z}$. $\;B_{z}>D_{y}$, $\frac{\partial V_{Q_{2}}}{\partial D_{x}}=-\frac{\left(B_{z}+D_{y}\right)\left(B_{z}-D_{y}\right)}{2D_{y}}*\frac{1}{D_{x}{}^{2}}<0$. The probability of local governments choosing subsidies is negatively correlated with corporate preferences. $\frac{\partial V_{Q_{2}}}{\partial D_{y}}=-2D_{y}*2D_{y}D_{x}-\left(B_{z}{}^{2}-D_{y}{}^{2}\right)*2D_{x}=-2D_{x}\left(D_{y}{}^{2}+B_{z}{}^{2}\right)<0$. The probability of local governments choosing subsidies is negatively correlated with farm household subsidy expenditures. The more the local government needs to spend financially, the lower the probability that the government chooses to subsidize. $\frac{\partial V_{Q_{2}}}{\partial B_{z}}=\frac{B_{z}}{D_{y}D_{x}}>0$, the higher the ecological cost borne by the local government, the higher the probability of choosing to subsidize it. □

Corollary 3 suggests that subject to local government performance and financial pressures, unilateral reliance on local government financial expenditures to sustain rural straw-returning behavior is not sustainable [37]. On the one hand, we need to improve local government performance assessment standards to include environmental management, increase ecological costs, and force local governments to pay attention. On the other hand, we need to actively mobilize the enthusiasm of market players and release market vitality. In this process, we should protect the interests of planting farmers, enterprises, and other subjects and realize the value-added of diversified market subjects [38].

### 3.4. Equilibrium Point and Stability Analysis of the Three-Party Evolutionary Game System

We let F(x)=0, F(y)=0*,* F(z)=0 to derive 8 pure strategic equilibria.$\;E_{1}=\left(0,0,0\right)$, $E_{2}=\left(1,0,0\right)$, $E_{3}=\left(0,1,0\right)$, $E_{4}=\left(0,0,1\right)$, $E_{5}=\left(1,1,0\right)$, $E_{6}=\left(1,0,1\right)$, $E_{7}=\left(0,1,1\right)$, $E_{8}=\left(1,1,1\right)$. The Jacobi matrix of the three-party evolutionary game system is

$$\begin{array}{ll} A=\left[\begin{array}{c} A_{1}\;A_{2}\;A_{3}\\ A_{4}\;A_{5}\;A_{6}\\ A_{7}\;A_{8}\;A_{9} \end{array}\right] & =\left[\begin{array}{c} \begin{array}{cc} \frac{\partial F\left(x\right)}{\partial x} & \frac{\partial F\left(x\right)}{\partial y}\;\frac{\partial F\left(x\right)}{\partial z}\\ \frac{\partial F\left(y\right)}{\partial x} & \frac{\partial F\left(y\right)}{\partial y}\;\frac{\partial F\left(y\right)}{\partial z} \end{array}\\ \frac{\partial F\left(z\right)}{\partial x}\;\frac{\partial F\left(z\right)}{\partial y}\;\frac{\partial F\left(z\right)}{\partial z} \end{array}\right]\\ & =[\begin{array}{ccc} \left(2x-1\right)\left(C_{1}-zD_{X}-C_{2}-B_{x}\right) & 0 & x\left(1-x\right)D_{x}\\ y\left(1-y\right)V_{2} & \left(2y-1\right)\left(2E-B_{y}-xV_{2}-zD_{y}\right) & y\left(1-y\right)D_{y}\;\\ z\left(z-1\right)D_{X} & z\left(z-1\right)D_{y} & \left(2z-1\right)(xD_{X}+yD_{y}-B_{z} \end{array}] \end{array}$$

According to the stable strategy judgment method of evolutionary games, Liapunov’s first method: if all the eigenvalues of the Jacobi matrix are negative, the equilibrium point is asymptotically stable. If at least one of the eigenvalues is positive, the equilibrium point is non-asymptotically stable. The stability analysis of the evolutionary game is shown in Table 4.

**Corollary 4.**  $\;B_{z}-D_{y}-D_{x}>0$, $2E-D_{y}-B_{y}-V_{2}<0$, $C_{1}-B_{x}-C_{2}-D_{x}<0$, there exists a stable point $E_{8}=\left(1,1,1\right)$ for the replication dynamics.

**Proof 4.**  As shown in Table 2, the conditions for satisfying Corollary 4, $D_{x}-B_{z}+D_{y}<0$. The hypothesis mentions that $B_{y}-2E<0$, $C_{1}-C_{2}>B_{x}$, $B_{z}>D_{x}$, $B_{z}>D_{y}$. We substitute these equations and arrive at the equilibrium point $E_{8}=\left(1,1,1\right)$, which is the only one that satisfies the terms as negative. Therefore, in this condition, there exists only one stable point $E_{8}=\left(1,1,1\right)$. □

Corollary 4 suggests that the cost of subsidies given by local governments to cultivating farmers and enterprises should be less than the ecological cost borne by local governments to effectively prevent the game system from having a stable combination of strategies in which farmers are willing to participate, enterprises are willing to produce, and governments are unwilling to subsidize. We have to guarantee that the benefits that enterprises obtain are greater than the costs to effectively prevent the gaming system from a stable combination of strategies in which farmers are willing to participate, enterprises are unwilling to produce, and the government is willing to subsidize. We need to guarantee that the subsidies and benefits received by planting farmers participating in straw return are greater than the costs to effectively prevent the gaming system from a stable combination of strategies in which farmers are unwilling to participate, companies are willing to produce, and the government is willing to subsidize.

## 4. Simulation Analysis

### 4.1. Dynamic Evolution of the Game of Behavior of Three Subjects in the Case of Vested Interests

**Figure 4:**
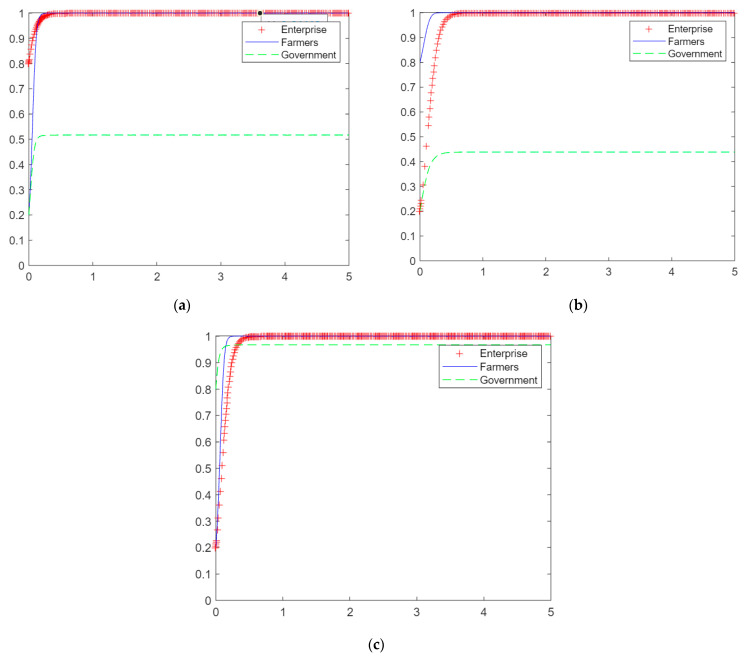
The dynamic evolution of the game of behavior of the three subjects in the case of vested interests. (**a**) Enterprise production, (**b**) farmers’ participation, (**c**) government subsidies.

To study the effect of single-subject behavior on the dynamic evolution of the straw utilization system, we suppose a set of values.$\;R_{p}=100$, $C_{1}=15$, $C_{2}=10$, $D_{X}=5$, $B_{x}=15$, $V_{1}=30$, $V_{2}=30$, $D_{y}=25$, E=10, $B_{y}=15$, A=60, $B_{z}=30$. We determine that each subject benefits from participating in the straw utilization system and analyze the results of single-subject behavior on the dynamic evolution of the system. The horizontal axis of Figure 4 represents time, and the vertical axis represents probability.

At the values set above, the benefits of the firm’s choice of production, the farmer’s choice of participation, and the government’s choice of subsidy strategy are all greater than those of no valorizing, no participation, and no subsidy. Assume that *x* = 0.8, *y* = 0.2, and *z* = 0.2, i.e., the probability of the game strategy of the firm choosing production is higher. The probability that growers choose to participate under the dynamic replication equation converges to 1, and the probability that local governments choose to subsidize does not converge to 1. Figure 4a shows that under the active production behavior of enterprises, growing farmers eventually choose to participate in straw return after the game and evolution, and local governments eventually choose not to subsidize after the game and evolution.

At the same values, the benefits of participation in the system are greater for the three subjects than nonparticipation, assuming *x* = 0.2, *y* = 0.8, and *z* = 0.2, i.e., when the plantation farmers choose to participate in the behavioral game strategy with a higher probability. Under the action of the dynamic replication equation, the probability of firms choosing to produce converges to 1, and the probability of local governments choosing to subsidize does not converge to 1. Figure 4b shows that with the active behavior of the grower farmers, the firms eventually choose to produce after the game and evolution, and the local government chooses not to subsidize. The active participation of growing farmers leads to an increase in the collection of straw on the market, with supply exceeding demand. This reduces procurement costs for companies and attracts them to choose production. However, it also shows that relying on the active participation of the farmer side is not conducive to the robust operation of the system.

At the same values, the benefits of participation in the system are greater for the three actors than nonparticipation, assuming *x* = 0.2, *y* = 0.2, and *z* = 0.8, i.e., when the local government has a higher probability of choosing the subsidy game strategy. Under the dynamic replication equation, the probabilities that firms, growers, and local governments choose to participate in the system all converge to 1. Figure 4c shows that with the positive behavior of local government subsidies, firms and growers eventually choose to produce and participate after the game and evolution. Under the active guidance of the local government, it can make the market profitable for all subjects and segments, and it can also make lasting profits for the government, which can promote the sound operation of the system.

### 4.2. Dynamic Evolution of the System’s Three-Subject Behavior Game under the Behavior of a Given Subject

**Figure 5:**
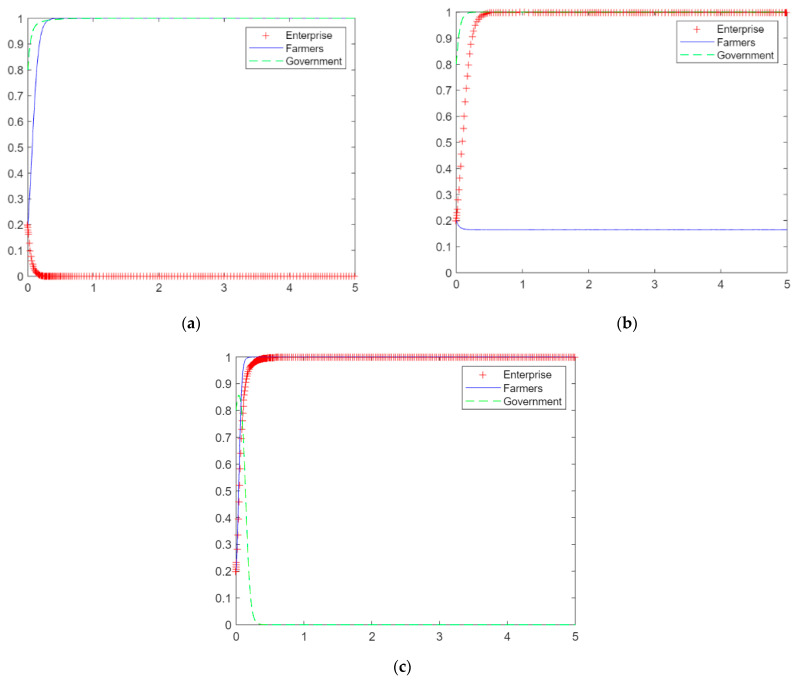
The dynamic evolution of the system’s three-subject behavior game under the behavior of the given subject. (**a**) Enterprise losses, (**b**) farmers’ losses, (**c**) government losses.

In the above analysis, we found that active government participation in the system can significantly contribute to the stable operation of the system, so it is assumed that *x* = 0.2, *y* = 0.2, and *z* = 0.8. With a high probability that the local government chooses the subsidy game strategy, we analyze the results of the dynamic evolution of each subject’s gains and losses on the system’s tripartite behavior game. Suppose a set of values is given, $\;R_{p}=100$, $C_{1}=50$, $C_{2}=10$, $D_{X}=5$, $B_{x}=15$, $V_{1}=30$, $V_{2}=30$, $D_{y}=25$, E=10, $B_{y}=15$, A=60, $B_{z}=30$. Figure 5a shows that firms benefit less from choosing to produce than from not producing, farmers benefit more from choosing to participate than from not participating, and local governments benefit more from choosing to subsidize than from not subsidizing. The probabilities of farmers choosing to participate and local governments choosing to subsidize converge to 1 under the dynamic replication equation, and the probability of firms choosing to produce does not converge to 1. This shows that with the active participation of local governments, enterprises choose to produce at a loss and eventually choose not to produce after the game and evolution, while farmers still get the benefit of government subsidies in straw return and eventually choose to participate after the game and evolution.

Given a set of data, $\;R_{p}=100$, $C_{1}=15$, $C_{2}=10$, $D_{X}=5$, $B_{x}=15$, $V_{1}=20$, $V_{2}=0$, $D_{y}=25$, E=20, $B_{y}=15$, A=60, $B_{z}=30$. Growing farmers benefit less from choosing to participate than from not participating, and companies and local governments benefit more from participating in the system than from not participating. With a high probability that the local government chooses the game strategy of subsidy, we analyze the results of the dynamic evolution of the game of each subject’s gain or loss on the system’s tripartite behavior. Figure 5b shows that under the dynamic replication equation, the probability of growers choosing to participate does not converge to 1, and the probability of firms choosing to produce and the government choosing to subsidize converge to 1. This indicates that with the active participation of local governments, enterprises are profitable and eventually choose to produce after gaming and evolution. However, farmers will encounter behaviors such as corporate reneging and not having more leisure to collect straw, resulting in farmers not receiving satisfactory benefits and eventually choosing not to participate after the game and evolution.

Given a set of data, $\;R_{p}=100$, $C_{1}=15$, $C_{2}=10$, $D_{X}=20$, $B_{x}=15$, $V_{1}=30$, $V_{2}=30$, $D_{y}=40$, E=10, $B_{y}=15$, A=20, $B_{z}=30$. Local governments benefit less from choosing to subsidize than from not subsidizing, and enterprises and growers benefit more from participating in the system than from not participating. With a high probability that the local government chooses the game strategy of subsidy, we analyze the results of the dynamic evolution of the game of each subject’s gain or loss on the system’s tripartite behavior. Figure 5c shows that under the dynamic replication equation, the probability that the local government chooses to subsidize does not converge to 1, and the probability that firms and growers choose to participate in the system converges to 1. This shows that if the subsidy given by the local government is greater than the benefit from it, the game and evolution eventually choose not to subsidize it. Growers and enterprises can benefit from the system and therefore eventually choose to participate in the system after the game and evolution.

## 5. Conclusions

Considering the different interests of enterprises, farmers, and local governments, this paper constructs a three-party game model among enterprises, planting farmers, and local governments to analyze the stability of straw return strategy selection and the influential relationships of each element. We provide policy suggestions for the construction of a multifaceted collaborative straw comprehensive utilization system based on the influential relationships of these factors and the dynamic evolution of the stability game.

In the section on simulation analysis, it is found that the game system does not converge to 1 when only enterprises or farmers participate in the straw return activities, and the game system converges to 1 when the government participates in the straw return activities. This shows that government power cannot be missing in the management of straw returns. In the corollaries, the study found that the reason for the low motivation of companies to participate in straw return is the high cost of processing the straw. The reason for the low motivation of farmers is that the leisure of farmers during the busy season is very expensive. Local governments are not motivated because ecological management is not included in the performance assessment.

We open the discussion on the governance of straw return by examining the results of the game between companies, farmers, and local governments. Due to long-term wrong farming experience, farmers fail to recognize the significance of straw return to farmland ecology, leading them to choose straw burning. Local governments should set up expert advocacy groups to organize policy advocacy, skills training, and trial demonstrations in various forms of activities during agricultural leisure time. Farmers actively participate in straw return activities, but they are limited by technology and operate incorrectly, jeopardizing the cultivation of the next crop and reducing farmers’ returns. Farmers’ willingness to participate in straw return activities is gradually decreasing. We need to make use of enterprises as market players to outsource the technical difficulties of straw return to the fields to enterprises and optimize resource allocation.

## Figures and Tables

**Table 1:** Game study of environmental stakeholders.

Research Theme	Game Subjects	Research Methodology	Research Findings	Reference
Game Theory	the central government and local governments	Evolutionary game model for both sides	The supervision of the central government is in direct proportion to the probability of local government violations.	[27]
	farmers and local governments	Evolutionary game model for both sides	The ecological protection behavior of farmers is affected by the probability of violation by local governments.	[28]
	ecological suppliers and ecological consumers	Evolutionary game model for both sides and CVM	No matter whether the ecological supplier chooses to protect or not, the ecological consumer chooses not to compensate.	[29]
	industrial management department, the technology promotion department, and the farmers	DEA—HR model	The collaborative innovation ability of the participants in the matrix recycling of agricultural waste is in a “coordinated and generally effective” state.	[30]
	government, farmers, and contractors	Three-way evolutionary game model	The government plays a guiding role in the behavior strategies of farmers and contractors, and farmers’ transfer of farmland is a prerequisite for the system to become stable; the farming cost is also an important factor affecting the three parties to reach a stable equilibrium point.	[31]
	the eastern, central, and western regions	Evolutionary game model for both sides	The eastern region took the lead in implementing farmland nonagricultural transformation, which caused pressure and difficulties for farmland protection in the central and western regions.	[32]

**Table 2:** Parameter symbols and their meanings.

Parameter	Meanings	Parameter	Meanings
$R_{p}$	Corporate income	$V_{2}$	Income received by farmers from the sale of straw
$C_{1}$	Cost of straw processing for companies	E	Farmers at leisure
$C_{2}$	Costs for companies not processing straw	$B_{y}$	Ecological costs are borne by farmers
$D_{x}$	Companies receive government subsidies	A	Government performance gains
$B_{x}$	Ecological costs are borne by companies	$B_{z}$	Ecological costs are borne by the government
$D_{y}$	Farmers receive government subsidies	$V_{1}$	Farmers’ income from normal operations

**Table 3:** Pure strategic gaming options for firms, growers, and local government.

	Growing Farmers		Government	
			Subsidies (*z*)	No Subsidy (1 − *z*)
firms	Valorizing (*x*)	Participation (*y*)	*R − C*_1_ *+ D_x_, V*_1_ *+ V*_2_ *+ D_y_ − E, A – D_x_ − D_y_*	*R − C*_1_ *, V*_1_ *+ V*_2_ *− E, A − B_z_*
		No participation (1 *− y*)	*R − C*_1_ *+ D_x_, V*_1_ *+ E − B_y_, A − D_x_ − B_y_*	*R − C*_1_ *, V*_1_ *+ E − B_y_, A − B_y_ − B_z_*
	No valorizing (1 *− x*)	Participation (*y*)	*R − C*_2_ *− B_x_, V*_1_ *+ D_y_ − E, A − D_y_ − B_x_*	*R − C*_2_ *− B_x_, V*_1_ *− E, A − B_x_ − B_z_*
		No participation (1 *− y*)	*R − C*_2_ *− B_x_, V*_1_ *+ E − B_y_, A − B_x_ − B_y_*	*R − C*_2_ *− B_x_, V*_1_ *+ E − B_y_, A − B_x_ − B_y_ − B_z_*

**Table 4:** Equilibrium point stability analysis.

Balancing Point	Jacobi Matrix Eigenvalues	Symbols	Stability
	$\lambda_{1},\;\lambda_{2},\lambda_{3}$		
(0,0,0)	*B_z_, B_y_ – 2 × E, B_x_ − C*_1_ *+ C*_2_	(+,−,−)	Unstable
(1,0,0)	*B_z_ – D_x_, B_y_ – 2 × E + V*_2_ *, C*_1_ *− B_x_ − C*_2_	(*,−,+)	Unstable
(0,1,0)	*B_z_ − D_y_, 2 × E − B_y_, Bx − C*_1_ *+ C*_2_	(+,+,−)	Unstable
(0,0,1)	*−B_z_, B_y_ + D_y_ – 2 × E, B_x_ − C*_1_ *+ C*_2_ *+ D_x_*	(−,+,+)	Unstable
(1,1,0)	*2 × E − B_y_ − V*_2_ *, C*_1_ *− B_x_ − C*_2_ *, B_z_ − D_x_ − D_y_*	(*,+,+)	Unstable
(1,0,1)	*D_x_ − B_z_, C*_1_ *− B_x_ − C*_2_ *− D_x_, B_y_ + D_y_ – 2 × E + V*_2_	(−,−,+)	Unstable
(0,1,1)	*D_y_ − B_z_, 2 × E − D_y_ − B_y_, B_x_ − C*_1_ *+ C*_2_ *+ D_x_*	(−,−,+)	Unstable
(1,1,1)	*D_x_ − B_z_ + D_y_, C*_1_ *− B_x_ − C*_2_ *− D_x_, 2 × E − D_y_ − B_y_ − V*_2_	(−,−,−)	ESS

Note: * indicates that the symbol is uncertain.

## Data Availability

The data underlying the results presented in the study are all available. The data presented in this study are available on request from the corresponding author. The data are not publicly available due to privacy.
